# Advancing Pancreatic Cancer Surgical Treatments and Proposal of New Approaches

**DOI:** 10.3390/cancers16162848

**Published:** 2024-08-15

**Authors:** Viviana Cortiana, Harshitha Vallabhaneni, Jade Gambill, Soumiya Nadar, Kennedy Itodo, Chandler H. Park, Yan Leyfman

**Affiliations:** 1 Department of Medical and Surgical Sciences (DIMEC), University of Bologna, 40126 Bologna, Italy; 2 Apollo Institute of Medical Sciences and Research, Hyderabad 517001, India; 3 Parker University, Dallas, TX 75229, USA; 4 Tbilisi State Medical University, 0186 Tbilisi, Georgia; 5 Nigerian Institute for Trypanosomiasis Research Jos, Kaduna PMB 2077, Nigeria; 6 Norton Cancer Institute, Louisville, KY 40202, USA; chandler.park@louisville.edu; 7 Icahn School of Medicine at Mount Sinai South Nassau, Oceanside, NY 11572, USA; yan.leyfman@mssm.edu

**Keywords:** pancreatic cancer, adenocarcinoma, borderline resectable pancreatic cancer, neoadjuvant therapy, surgical anatomy, vascular involvement, multidisciplinary approach

## Abstract

**Simple Summary:**

Pancreatic cancer is a significant challenge due to its aggressive nature, complex management, high mortality rate, and low 5-year survival rate. Approximately 85% of cases are adenocarcinomas, while endocrine tumors comprise less than 5%. Borderline reresections andcally advanced pancreatic cancers are difficult to treat due to frequent vascular involvement, complicating complete resections, and increasing morbidity. Various therapeutic modalities aim to improve patient outcomes. Traditionally, upfront surgery was the standard for resectable tumors, with multimodal chemotherapy being central to treatment. Understanding surgical anatomy is pivotal for enhancing outcomes. Classification systems like the MD Anderson Criteria assess tumor involvement with major blood vessels. Neoadjuvant therapy addresses micro-metastatic disease early, increasing R0 resection chances. Dr. Sanjay S. Reddy’s insights highlight the importance of a multidisciplinary approach to advancing therapy and improving prognosis.

**Abstract:**

Pancreatic cancer is a significant challenge in oncology due to its aggressive nature and complex management, leading to high mortality rates and a dismally low 5-year survival rate. Approximately 85% of cases manifest as adenocarcinoma, while endocrine tumors constitute less than 5%. Borderline resectable and locally advanced pancreatic cancers are particularly difficult to treat due to vascular involvement, which complicates complete resections and increases morbidity. Various therapeutic modalities aim to overcome these challenges and improve patient outcomes. Traditionally, upfront surgery was the standard for resectable tumors, with multimodal chemotherapy being central to treatment. Understanding surgical anatomy is pivotal in enhancing surgical outcomes and patient survival. Resectability challenges are several when seeking to achieve R0 resections, particularly for borderline resectable tumors. Various classification systems—the MD Anderson criteria, the NCCN criteria, the AHPA/SSAT/SSO consensus statement, and the Alliance definition—assess tumor involvement with major blood vessels, with the first of these systems being broadly accepted. Vascular staging integration is also important, with the Ishikawa staging system using preoperative imaging to assess venous involvement. Furthermore, neoadjuvant therapy enhances treatment effectiveness by addressing micro-metastatic disease early, increasing R0 resection chances, and downstaging tumors for optimal surgery. Insights from the Fox Chase Cancer Center’s neoadjuvant treatment approach highlight the importance of a multidisciplinary strategy when advancing therapy and improving patient prognosis. This commentary, inspired by Dr. Sanjay S. Reddy’s Keynote Conference during MedNews week, highlights current advancements and ongoing challenges in the treatment of pancreatic cancer, emphasizing the need for a comprehensive, multidisciplinary approach to improve outcomes.

## 1. Introduction

The pancreas is an organ with a significant and dual role, being part of both the digestive and endocrine systems [1]. Malignancy in the pancreas, unfortunately, is not uncommon [2]. The pancreas is anatomically divided into four separate parts: the head, the neck, the body, and the tail [1]. Their numerous relations are crucial for surgeons when considering resectability. In particular, the superior mesenteric artery (SMA) is crucial in pancreatic cancer management decisions and runs right through the pancreas, between the tail and neck area of the pancreas [1,2]. As highlighted by MedNews week’s keynote speaker, Dr. Sanjay Reddy, during his presentation, *GI Oncology Update: Pancreatic Cancer*, this key artery can indeed often cause complications when determining treatment options for pancreatic cancer patients [2].

Pancreatic cancer is the 11th most common cancer, with an incidence rate of 12.6 per 100,000 men and women annually and a mortality rate of 10.9 per 100,000 [2]. Approximately 1.6% of people will be diagnosed with pancreatic cancer in their lifetime. In 2015, an estimated 68,615 individuals were living with pancreatic cancer in the United States [2]. Recent statistics indicate that pancreatic cancer accounts for 3% of all cancers in the United States and 7% of all cancer-related deaths [3]. Although these figures are lower compared with some other cancers, they are still alarming, especially given the high mortality of the disease, and therefore highlight the need for greater attention and research.

Pancreatic cancer is notoriously difficult to diagnose early due to its asymptomatic nature in the initial stages and the lack of effective screening methods. This often leads to a diagnosis only after the cancer has progressed to an advanced and inoperable stage. Consequently, the prognosis for pancreatic cancer patients remains poor. Despite these challenges, there have been some minor improvements in survival rates [4]. Encouragingly, the 5-year relative survival rate for pancreatic cancer has shown a slow but steady increase, rising from 8.5% during 2008–2014 to 12.8% during 2014–2020 [5]. This modest rise reflects a slightly more optimistic outlook for patients diagnosed with pancreatic cancer. However, approximately 52% of patients are still diagnosed at an advanced stage where the cancer has already metastasized, significantly impacting overall survival rates [2,5]. As with all cancers, the survival rates decrease markedly when identified at later stages [2].

This commentary aims to explore the complexities and challenges of pancreatic cancer, with a particular focus on recent advancements in vascular staging, neoadjuvant trials, and emerging therapies. We will present and discuss the potential of these new treatment modalities to improve survival rates, provide a better prognosis for patients and address the currently grim 5-year relative survival rate. By highlighting innovative studies and assessing the efficacy of these new and combined therapies, we hope to provide insights into improving outcomes for pancreatic cancer patients.

## 2. Navigating the Gray Area: Unraveling the Complexity of Resectability in Pancreatic Cancer

When looking at pancreatic cancer, survival rate odds are significantly impacted by the cancer’s resectability. A successful pancreatoduodenectomy, or surgical removal of the mass, dramatically improves survival rates. Most patients present with either locally advanced or metastatic cancer; therefore, only 20–25% of patients have potentially resectable cancer at diagnosis [6]. If the cancer is successfully resected, the patient’s 5-year survival rate increases to approximately 15–20%, a substantial improvement compared with non-resectable cases. Unfortunately, those who undergo only a partial, margin-positive resection tend to have poor outcomes, comparable to those with locally advanced cancer [2]. These drastic differences in survival outcomes highlight the importance of clear guidelines for determining resectability.

Resectability status is categorized into resectable, borderline resectable, and locally advanced, non-resectable cancers. According to the guidelines of the National Comprehensive Cancer Network (NCCN), borderline resectable pancreatic cancer typically involves the tumor probably touching or slightly invading the superior mesenteric artery, celiac artery, or portal vein, but without causing significant narrowing or blockage and with no distant metastases.

However, these guidelines can be influenced by the operating surgeon’s bias and comfort level, as the procedure near the superior mesenteric artery carries high risks.

Borderline resectable pancreatic tumors are further classified into three types: A, B, and C [2]. Type A involves the tumor abutment, encasing less than 180 degrees of the superior mesenteric artery’s circumference, or involving a small segment of the hepatic artery or a reconstructible short segment occlusion of the superior mesenteric vein/portal vein [2,7,8]. Type B includes the same criteria as type A but with the possibility of extrapancreatic metastatic cancer [2,7,8]. Type C encompasses the criteria of type A but includes patients with marginal performance status or severe pre-existing medical comorbidities.

Incorporating advancements in surgical techniques and patient selection criteria can further improve the resectability and outcomes of pancreatic cancer surgeries. Enhanced imaging technologies and personalized treatment plans are increasingly playing a role in determining the best surgical approach for each patient. Through ongoing research and clinical trials, the medical community continues to refine these guidelines and develop new strategies by which to increase the success rates of pancreatic cancer surgeries and improve overall patient outcomes.

## 3. Integrating Vascular Staging into Pancreatic Cancer Management

Patients diagnosed with pancreatic cancer are often divided into one of four categories based on the extent of the disease: resectable, borderline resectable, locally advanced, and metastatic [9]. The stage of disease that is referred to as borderline resectable pancreatic cancer has gained interest and is now the focus of several multi-institutional clinical investigations [10].

The concept of borderline resectable pancreatic cancer has developed from various clinical observations gathered over decades. However, there is no consensus regarding the definition of borderline resectability in pancreatic adenocarcinoma. The National Comprehensive Cancer Network characterizes a tumor in the head or body of the pancreas as borderline resectable if there is severe impingement of the portal and/or superior mesenteric vein (PV-SMV), ≤180° abutment of the superior mesenteric artery (SMA), reconstructible abutment or encasement of the hepatic artery, or reconstructible SMV occlusion [8,11]. A recent consensus conference developed the following criteria for borderline resectable PV-SMV involvement: tumor-associated deformity, ≥180° abutment, or reconstructible short segment occlusion [12]. Thus, the degree of venous involvement that constitutes borderline resectability is not clearly defined, ranging from impingement to occlusion.

**Figure 1:**
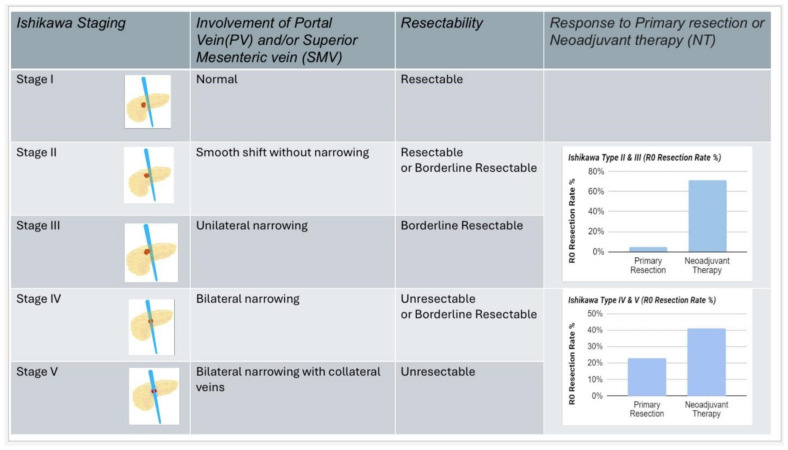
Evaluating Preoperative Therapy Effectiveness in Pancreatic Cancer Across Various Resectability Criteria [10].

One study was conducted to identify the involvement of PV-SMV and to determine which patients benefit from preoperative therapy [13]. Venous involvement was classified by preoperative computed tomography according to Ishikawa types, as follows: type I: normal; type II: smooth shift/displacement with normal caliber; type III: unilateral narrowing; type IV: bilateral narrowing; and type V: bilateral narrowing with collateral formation [14]. Preoperative chemoradiation was found to be associated with a higher R0 resection rate and negative lymph nodes (both *p* < 0.0001) but did not affect the need for vein resection. When classified by Ishikawa types, preoperative therapy significantly improved R0 resection among patients with types II and III that otherwise have low R0 resection rates with primary resection when compared with that of types IV and V [13]. Similarly, the correlation between preoperative therapy and R0 resection rate was observed only among patients with Ishikawa types II and III. In patients with Ishikawa type II and III tumors, preoperative therapy was associated with higher margin-negative resection and survival rates. Patients with bilateral venous narrowing were less likely to benefit from preoperative treatment [13] (Figure 1).

Another study, concluded in 2013, investigated the utility of preoperative vascular grading in patients undergoing surgery first for pancreatic cancer, particularly focusing on whether radiologic arterial or venous involvement can predict pathologic margin status [15]. They adopted the Ishikawa classification system in order to grade venous involvement and categorized arterial involvement based on preoperative imaging. For patients with both classifications recorded, vascular involvement was categorized as “None”, “Arterial only”, “Venous only”, or “Both” and the association of vascular involvement and pathologic margin status was examined [15]. The study found that Ishikawa grading was strongly associated with a positive superior mesenteric artery (SMA) and superior mesenteric vein (SMV) margin, while arterial staging showed no association with SMA or SMV margin. These findings suggest that Ishikawa grading is more predictive of arterial involvement and remains significant in multivariate analysis [15]. The study also discusses the historical context of pancreatic cancer resectability criteria and the importance of accurate radiographic imaging in treatment planning [12]. This highlights the need for criteria that define tumor resectability accurately, especially for borderline resectable tumors, to improve the rate of complete resection and overall patient outcomes.

The classification and management of borderline resectable pancreatic cancer remain complex and evolving. Despite various definitions and criteria from different institutions, there is still no definitive and universal consensus on what constitutes borderline resectability.

Despite these advances, the field still faces significant challenges. The lack of standardized definitions and criteria for borderline resectability leads to variations in clinical practice and can impact patient outcomes. Therefore, further research is essential in order to refine these criteria and develop a consensus on the optimal management strategies for patients with borderline resectable pancreatic cancer. Studies have highlighted the importance of precise criteria in radiographic evaluation to improve the complete resection rates and overall patient outcomes. This precision is particularly relevant for borderline resectable tumors. Multicenter studies and collaborative efforts are therefore needed and awaited to validate existing classification systems and potentially integrate them into a unified framework that can guide clinical decision making.

## 4. Charting the Course: Neoadjuvant Trials in Pancreatic Cancer Research

Clinical trials are crucial phases in the development of neoadjuvant therapy for pancreatic cancer, providing emerging data to support its use. Traditionally, the standard approach has involved adjuvant chemotherapy, where patients undergo surgery followed by chemotherapy. However, data such as that of the NeoALTTO trial [16] have revealed a significant issue: many patients are unfit to receive chemotherapy after surgery. This has led to the consideration of administering therapy beforehand to maximize its potential benefits. This was further investigated in the NORPACT-1 and NORPACT-2 trials, which assessed the effects of neoadjuvant therapy (chemotherapy before surgical resection) and adjuvant chemotherapy (chemotherapy after surgical resection) in patients with resectable and borderline resectable pancreatic cancer, respectively. Both trials demonstrated a higher rate of R0 resection associated with neoadjuvant therapy. While the NORPACT-1 trial did not show a significant improvement in overall survival with neoadjuvant therapy, the NORPACT-2 trial suggested potential benefits in overall survival, though these findings require further validation.

Neoadjuvant therapy provides several key advantages. It offers a period to assess the cancer’s aggressiveness, allowing observation of the disease’s biology. It also treats micro-metastatic disease early and aims to downstage the tumor to achieve an R0 resection, which is the goal of completely removing the tumor with no residual microscopic or macroscopic disease.

In the 1990s, Doug Evans, a mentor to many contemporary surgeons, pioneered a novel approach to pancreatic cancer. His study [17], Preoperative Chemoradiation and Pancreaticoduodenectomy for Adenocarcinoma of the Pancreas, was the first to describe pancreatic cancer as a “potentially resectable disease”. This single-institution study involved 28 patients who received chemoradiation for 5.5 weeks before surgery. Contrary to the prevailing belief, all 28 patients were fit enough for surgery post-chemotherapy. The toxicity levels were acceptable, and the study introduced a grading system for the radiation treatment effect. Dr. Sanjay and his group found that higher tumor kill rates correlated with higher survival rates and that margin-negative resections were significantly better for these patients. The study’s results therefore indicate that preoperative chemoradiation is safe and feasible, increasing the likelihood of patients receiving full treatment and potentially enhancing radiation effectiveness by targeting well-oxygenated cells before surgical devascularization.

Following these promising results, Dr. John Hoffman conducted a pilot study [18] at the Fox Chase Center, hypothesizing that administering neoadjuvant radiotherapy preoperatively with chemotherapy could enhance local control. His study showed a remarkable median survival time of 45 months from diagnosis, demonstrating the safety and tolerability of this approach for localized pancreatic cancer. This subsequently led to a phase II trial [19] examining preoperative radiation therapy and chemotherapy for localized resectable adenocarcinoma of the pancreas. The RT target volumes were determined using CT scans and/or surgically placed markers. The initial target volume was a tumor with a 3 cm margin, while the boost (conedown) target volume was a tumor with a 2 cm margin. Custom blocking material with at least five half-value layers of dose attenuation was used to shield normal tissues and define target volumes. Dose homogeneity within ±5% was required. Margins were adjusted as needed to meet normal tissue tolerance limits. The initial target volume was specified to be treated to a dose of 3960 cGy, and the boost target volume was specified to be treated to a dose of 1080 cGy, for a total dose of 5040 cGy. Treatments were delivered through three or four fields using 180 cGy fractions once daily, five days a week, on megavoltage equipment with photon energy of 4 MV or greater. At least 50% of the functional renal parenchyma was restricted to a dose of 2000 cGy or less, and the spinal cord dose was limited to 4000 cGy or less. Hepatic toxicity, resulting from tumor- or stent-related biliary obstruction or cholangitis, was as common and severe as any treatment-induced toxicity. There were two deaths from cholangitis following the completion of CTRT in patients with endoscopically placed stents, both of whom developed septicemia and cholangitis. One of these stents was removed and could not be replaced endoscopically. Among the 27 patients who experienced grade 3 to 5 liver toxicity, three had endoscopically placed stents; jaundice was corrected in two of these cases, and one was eventually corrected with a radiologically placed stent. Six patients were successfully treated with radiologically placed stents, and four experienced mild jaundice, either following initial cholecystojejunostomy (two cases) or without any intervention (two cases). Fourteen patients developed cholangitis, which was easily resolved in six patients with radiologically placed stents through stent changes and antimicrobials, and in one patient with a previous biliary bypass using antimicrobials alone. However, among the seven patients with endoscopically placed stents who developed cholangitis, only one experienced an easy resolution with antibiotics. Besides the two stent-related deaths, one patient needed an 11-day hospital stay with antibiotics, and three patients (including one with a stent-related hepatic abscess) required salvage with radiologically placed stents. Among the 56 patients with tumors in the pancreatic head area, prior biliary bypass, endoscopically placed stents, and radiologically placed stents were used in 17, 18, and 13 patients, respectively. The incidence of cholangitis was significantly lower in those with prior biliary bypasses (*p* = 0.022). While the incidence of cholangitis was similar between the groups with stents, severe and lethal infections occurred only in those with endoscopically placed stents (6 out of 18 cases, compared with 0 out of 13 cases, *p* = 0.011) [19]. The study also faced challenges due to the inclusion of patients with advanced tumors, highlighting the need for clear definitions of resectability in clinical trials. The study’s results suggest that the treatment plan might be more effective for patients with less advanced cancers.

In subsequent research, the FOLFIRINOX vs. Gemcitabine study [20], the FOLFIRINOX treatment involved administering oxaliplatin (85 mg/m^2^ of body-surface area), irinotecan (180 mg/m^2^), leucovorin (400 mg/m^2^), and fluorouracil (400 mg/m^2^ as a bolus, followed by 2400 mg/m^2^ as a continuous infusion over 46 h) every two weeks. Alternatively, gemcitabine was given at a dose of 1000 mg/m^2^ weekly for 7 out of 8 weeks, then weekly for 3 out of 4 weeks. The study showed that FOLFIRINOX significantly increased overall survival by 4.5 months compared with gemcitabine, despite causing more side effects. This led to a preoperative modified FOLFIRINOX study [21] followed by Capecitabine-based chemoradiation for borderline resectable pancreatic cancer [21], which demonstrated improved R0 resection rates and tumor kill rates.

The “Alliance for Clinical Trials in Oncology (ALLIANCE) trial A021501” [22] reported that chemotherapy followed by radiation therapy for borderline resectable pancreatic cancer resulted in favorable overall survival. This study emphasized the importance of disease definition and highlighted the potential for shorter radiation treatments to improve outcomes. The SWOG S1505 study conducted in 2010 [23] compared neoadjuvant chemotherapy to upfront surgery, showing no significant survival difference but indicating neoadjuvant chemotherapy’s benefits in resectable pancreatic cancer. This underscored the need for better methods by which to identify resectable patients and the safety and effectiveness of modern chemotherapy regimens before surgery. The PREOPANC trial [24] and the PROPANC-2 trial in 2021 [25] further support the use of neoadjuvant therapy for resectable and borderline resectable pancreatic cancer, demonstrating improved overall survival and treatment effectiveness.

Despite these advancements, some contrasting views in the field exist. One study [26] has shown that patients undergoing surgery first lived longer than those receiving neoadjuvant FOLFIRINOX, although this was influenced by study design aspects, such as a low completion rate of the assigned neoadjuvant chemotherapy due to side effects.

Cancer treatment can be categorized into two major approaches: those focused at locoregional control—generally surgical excision or radiation—and those aimed at systemic control—generally infusional therapy utilizing cytotoxic or, more recently, targeted chemotherapeutic agents [1]. Recent advancements in complementary locoregional therapies for cancer treatment include various innovative approaches such as intra-arterial chemotherapy and the combination of chemotherapy with external hyperthermia (HT) and radiotherapy (RT).

HT, also referred to as thermal therapy or thermotherapy, is a treatment method with the potential to work synergistically with other therapies. HT has a variety of effects on the body and has been recognized as a sensitizer for both RT and chemotherapy in multiple cancer types due to its diverse mechanisms of action [27,28]. This technique exploits the increased sensitivity of cancer cells to heat while aiming to reduce harm to nearby healthy tissues. HT can be categorized broadly based on the treatment area or the temperature used. Local HT typically involves the use of external energy sources, such as microwave, radiofrequency, ultrasound, or infrared devices, to heat the tumor area. Regional HT targets larger sections of the body, such as an entire limb or organ, using specialized heating devices or by perfusing heated fluids into the region. Whole-body HT involves heating the entire body and is used for treating metastatic cancer [29]. The temperatures used in HT vary, making temperature-based classification challenging. Lower temperatures, within the fever range, are generally associated with immune enhancement, while higher temperatures, around 43 °C, are intended for direct cell destruction or ablation [30].

Studies have identified mechanisms by which combining chemotherapy with HT can enhance antitumor effects, for example by increasing drug delivery to cancer cells and enhancing drug sensitivity in hyperthermic conditions. However, clinical research on combining intra-arterial hepatic (IHA) chemotherapy with HT is limited. Some non-controlled studies have shown therapeutic benefits when treating unresectable metastatic hepatic tumors with this combination.

A retrospective case-matched control study [2] involving 64 patients found that the group receiving combined treatment (group A) had a higher overall partial response (PR) rate and a lower overall progressive disease (PD) rate compared with the control group (group B). Although the survival benefit was not statistically significant, group A showed moderate prolongation of survival, especially in patients with metastatic gastric cancer. Enhanced evaluation methods like contrast enhancement CT might provide a more favorable assessment of therapeutic effects. Patients with liver metastases from gastric and colorectal cancers who responded to treatment in both groups had significantly longer survival than non-responders. Effective local control of liver metastases contributes to prolonged survival in patients without life-threatening extrahepatic metastases.

In treatments combining IHA chemotherapy with HT [3], mixed results were observed. While combining cisplatin (CDDP) and 5-fluorouracil (5-FU) with HT showed no enhanced antitumor effects, a combination of mitomycin C (MMC) and 5-FU with regional hyperthermia resulted in a 100% partial response rate. This suggests that the benefits of combining IHA chemotherapy with HT may be specific to certain drugs like MMC. Higher temperatures in hyperthermia treatments, ideally reaching 42 °C or higher, are thought to enhance antitumor effects, but achieving these temperatures consistently is challenging with current equipment. There was no close correlation found between maximum intratumor temperature and antitumor effects in the study group, likely due to uneven heat distribution within tumors.

One of the major challenges when treating advanced and metastatic hepatopancreatobiliary cancers is finding a regimen that improves therapy efficacy without adding adverse effects. Adjuvant HT meets these criteria, significantly enhancing local and overall treatment response, prolonging progression-free and overall survival, and improving laboratory results. Studies and case reports [5] have indicated that HT can also improve quality of life, with some patients returning to active lifestyles post-treatment. Although HT has adverse effects associated with local heating, these are generally mild (grade II complications).

Regional HT and modulated electro-hyperthermia (mEHT) are more advantageous and accepted than whole-body hyperthermia, as they do not require conscious sedation or long treatment times. HT’s precise clinical indication for disease stage remains unclear, and it is often used palliatively when traditional options are exhausted. Stratifying patients by tumor load, involved organs, and serum tumor marker levels could enhance treatment efficacy. Higher Eastern Cooperative Oncology Group performance status or increased body fluids may limit HT administration.

Overall, HT shows promise as a complementary treatment for advanced hepatopancreatobiliary cancers, however, while some therapeutic benefits were observed, prospective randomized studies are needed to accurately evaluate the effects of IHA chemotherapy with and without regional HT on unresectable hepatic tumors [31,32,33,34].

Therefore, neoadjuvant therapy remains an emerging and promising area of research in pancreatic cancer treatment, offering several advantages such as in the assessment of cancer aggressiveness and by potentially improving survival rates. While early research has shown promise, recent trials have provided mixed outcomes. Further research is needed and encouraged to determine the best treatment plans for individual patients.

## 5. Advancements in the Treatment of Pancreatic Ductal Adenocarcinoma: The Role of Total Neoadjuvant Therapy

Pancreatic cancer’s low overall survival rates due to frequent metastasis highlight the necessity for effective systemic therapies within a multimodal treatment framework [35,36]. Clinicians are therefore exploring various strategies, including chemotherapy (CT), chemoradiation (CRT), and total neoadjuvant therapy (TNT), to optimize patient outcomes and achieve complete responses. TNT, which integrates chemotherapy and radiotherapy, whether through induction TNT (chemotherapy preceding chemoradiotherapy) or consolidation TNT (chemoradiotherapy preceding chemotherapy), offers significant advantages over conventional treatment approaches. These include higher rates of tumor downstaging and pathologic complete response (PCR), which are critical factors for long-term survival, wherein no viable malignant cells are detected in the surgically resected specimen, consequently improving overall survival rates [36,37]. Patients achieving PCR are less likely to experience local tumor recurrence and have improved survival outcomes, with a median overall survival extending beyond 100 months, offering a potential for cure in this aggressive disease [36]. Additionally, TNT enhances compliance with planned therapeutic regimens by administering chemotherapy early, targeting occult micrometastases, and allowing for the assessment of chemosensitivity [36]. By reducing the tumor stage before surgery, TNT makes surgical resection more feasible and potentially less complex. Moreover, these decreases involved margins, effectively target micrometastatic disease and provide the opportunity to assess tumor biology before surgical intervention, thus potentially leading to disease downstaging. Furthermore, TNT increases the likelihood of achieving a complete pathologic response.

The innovative approach of TNT for pancreatic cancer is supported by recent clinical trials, such as SWOG S1505, the OPRA trial, the PRODIGE study, and the Alliance A021806 trial, which demonstrate the efficacy of neoadjuvant therapies in resectable pancreatic cancer [36]. The critical importance of accurate staging before every step of TNT is essential to avoid subjecting patients to surgery if they have either micrometastatic or overt metastatic disease at the time of surgery [2]. Proper staging ensures that only those patients who are most likely to benefit from surgical intervention proceed to this stage of treatment, thereby optimizing outcomes and avoiding unnecessary procedures. Dr. Sanjay Reddy, from Fox Chase Cancer Center (FCCC), provides an in-depth analysis of the various classifications and considerations involved in the clinical trials conducted at FCCC, highlighting the rigorous organization and strong evidence-based foundation of this research. Within these trials, the NCCN resectability criteria serve as the cornerstone, with patients’ clinical stage and CA-19-9 levels observed concurrently. A substantial proportion of patients categorized as borderline resectable or at an advanced clinical stage were assigned to the TNT group, aiming to improve their chances of survival. Notably, the data from FCCC indicated no significant correlation between CA-19-9 levels and patient outcomes, contrary to the data from other studies [2]. These results reflect the advantages brought by TNT over surgery-first, single-modality therapy, and adjuvant therapy groups, including better survival curves, with fibrosis observed in 80% of these patients compared with 70% in the single-modality group. Additionally, 65% of patients in the TNT group experienced tumor downstaging, with an impressive 86% achieving R0 resection. Furthermore, a 10% complete pathologic response was achieved in the TNT group, correlating with a median survival of 100.2 months for these patients. This also highlights a significant issue regarding compliance within the adjuvant therapy group, where 30% could not receive adjuvant therapy due to complications or physical debilitation [2].

**Figure 2:**
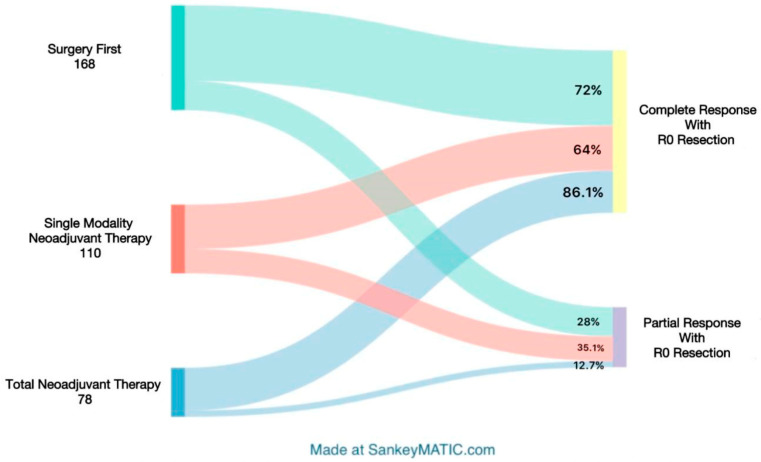
Mapping Patient Progress: Treatment Modalities to Final Outcomes in Pancreatic Cancer [36].

This clarifies and strengthens the rationale for including TNT and neoadjuvant therapies in the pancreatic cancer treatment regimen. Additionally, numerous studies have improved neoadjuvant therapy by combining immunotherapy, targeted therapies, and the use of predictive biomarkers (Figure 2). Among the targeted therapies are those that target tumor stem cells, improve drug delivery, alter the tumor microenvironment, and boost antitumor immunity [38]. Several genetic markers have been identified as prognostic and therapeutic markers by which to accurately determine the efficacy of treatment. These markers include homologous recombination repair deficiency (HRD), microsatellite instability, HER2/HER3, CDK4/6, NTRK fusions, KRAS G12C, and BRAF mutations, as well as germline BRCA mutations [39,40]. In addition to these, CA 19-9 and serial circulating tumor DNA (ctDNA) are also being investigated as prognostic markers [41]. Following these promising results, healthcare professionals are therefore encouraged to approach their research in a way that advances the goal of precision oncology as a whole.

## 6. Conclusions and Future Perspectives

Dr. Sanjay S. Reddy’s keynote provides an in-depth exploration of the comprehensive approach required for the effective management of pancreatic cancer, emphasizing several critical aspects that clinicians should consider. It begins by tracing the evolutionary trajectory of pancreatic cancer treatment, highlighting the contributions of collaborative efforts among clinicians from various regions and historical periods. This historical context highlights the progressive nature of current practices and the cumulative advancements that have shaped today’s treatment paradigms. Central to the commentary is the understanding that the survival rates of pancreatic cancer patients are heavily influenced by the resectability of the tumor. Procedures such as pancreatoduodenectomy (the Whipple procedure) are pivotal and achieving an R0 resection—where no cancer cells are seen at the resection margins—remains the gold standard. Positive margin resections are associated with significantly poorer survival outcomes. To address this, studies have shown that preoperative chemoradiation can enhance resection margins and survival rates, particularly in patients with less severe venous involvement (Ishikawa types II and III).

The commentary also discusses a range of treatment strategies aimed at achieving R0 resection. Dr. Sanjay evaluates the pros and cons of these strategies, advocating for active engagement in evidence-based interventions over a passive “watch and wait” approach. Accurate radiographic imaging and well-defined resectability criteria are highlighted as essential tools for effective treatment planning and improved patient outcomes, especially for those with borderline resectable tumors, and, in addition to surgical considerations, emerge as fundamental to adopting evidence-based approaches in clinical practice. This calls for uniformity in treatment protocols, diagnostic criteria, and patient management strategies, particularly as awareness and novel therapeutic modalities emerge. Consistent and well-informed clinical practices are vital for achieving optimal patient outcomes. Moreover, the need for rigorous and standardized clinical trials is addressed. Dr. Reddy strongly advocates for trials designed with evidence-based methodologies and predefined guidelines at each stage of treatment. Such trials are crucial for generating reliable data that can inform clinical practice and ensure the delivery of high-quality care to patients.

In conclusion, the commentary highlights the importance of a comprehensive and evidence-based approach to pancreatic cancer treatment. Accurate staging, well-defined treatment protocols, and standardized clinical trials are essential to improving patient outcomes. By advocating for consistent and informed clinical practices, the goal is a unified effort to advance the treatment and management of pancreatic cancer, ultimately aiming for improved survival rates and quality of life for affected patients.

## Data Availability

No patient data were directly utilized in this study.
